# Observations on Cases of Appendicitis

**Published:** 1902-12

**Authors:** T. Carwardine

**Affiliations:** Assistant Surgeon, Bristol Royal Infirmary


### OBSERVATIONS ON CASES OF APPENDICITIS.

BY

T. Carwardine, M.S. Lond., F.R.C.S.,

*Assistant Surgeon, Bristol Royal Infirmary.*

The following observations are based upon a consideration of 28 cases of operation for appendicitis: one half of which were performed for suppurative lesions, and the other half for the removal of the appendix in the interval between attacks. The patients all completely recovered except one who had pneumonia at the time of operation, but for whom it was decided in consultation that operation afforded the only chance. There were two separate intra-abdominal abscesses in this case, one in the loin and the other in the pelvis, separated by an adherent cæcum, and both were simultaneously drained. I am indebted to his medical attendant for the information that after making hopeful progress for a few days he suddenly developed septic meningitis.

The majority of operations for suppurative appendicitis were performed in private, whereas the majority of those for appendicectomy were performed in hospital practice. The average ages of the patients in the two classes were practically the same, viz., 30 and 31½ respectively; but the extremes differed n the two classes. The youngest patient with suppurative appendicitis was 9, and the oldest two 60 and 68; whereas the youngest for appendicectomy was 15, and the oldest two 48 and 66. In only half the cases of abscess had a previous attack of appendicitis been noted.

We may now consider some of the pathological peculiarities and complications met with.

**Appendicectomy for recurrent appendicitis.** The conditions which may be found cannot be determined beforehand with any degree of certainty. The appendix may be long or short straight or twisted or kinked; it may be free or buried in a mass of adhesions, or attached to various neighbouring structures; it may be a solid cord or distended by contents. Commonly the lumen is constricted at one part, and beyond this the mucous membrane is swollen and in contact with mucus or a fæcal concretion: on the other hand, the distal extremity may be quite occluded. Hemorrhages into the submucous tissue are frequent features. The position of the appendix varies widely: in one of the series it was to the outer side of the colon, twisted and adherent, close to the liver, the cæcum itself being turned up into the right loin; in another, a small appendix was found buried in firm adhesions in the sac of a femoral hernia. In three cases it was pelvic in position; and in one of these the appendix was long, passed over the iliac vessels, and its outer end was buried in a firm mass of adhesions in the deepest part of the pelvis behind the bladder, whence it could only be enucleated digitally without inspection. Once the organ was found embedded behind the cæcum; and in the remainder of the cases the appendix projected into the abdomen towards the middle line, either free or adherent to or incorporated with surrounding parts, such as the omentum, anterior parietes, etc. Adhesions were a marked feature in over half the cases; but they do not necessarily remain after previous attacks, for in two cases, with three and two previous attacks respectively, adhesions were absent. The presence of adhesions seems to depend partly on the anatomical position, whether confined or free, and partly on the amount of local peritonitis which accompanied the previous attacks. In half the cases the appendix was either markedly kinked or tortuous, and in one the appendix was so tortuous that when released from its attachments it twirled around as if it were alive. Any marked kink is usually accompanied by a clubbing and fixation of the distal end. In one case the appendix was apparently solid in its distal half, and in another case it was distended into a cyst and was complicated with the presence of a large ovarian cyst. The patients all recovered satisfactorily without any sequelæ, and in no case has the operation been followed by hernia or weakness of the scar.

**Suppurative Appendicitis.** This condition often gives rise to the greatest anxiety, and to the need of valour on the one hand and of discretion on the other. It is quite impossible to adopt any definite rule or to formulate a fixed creed for surgical action. Each case has to be judged on its merits, and the best guide is the formation of a mental picture of the processes which are taking place in the abdomen. The suppuration may place the patient in the greatest jeopardy within two days of the onset of an attack, or allow the patient to be comparatively comfortable at the end of a week or ten days. Moreover, there is often a delusive subsidence of the symptoms after the first few days which may act as a trap for the unwary: I refer to the fall of temperature, the softening of the pulse, and the diminution of prominence pain and tenderness which accompany the reduction of tension in an abscess when its area is increased. This is also frequently accompanied by the appear-

22

ance of resonance over an area previously dull, not the normal resonance, but the deceptive resonance of gas formation in the abscess cavity. As a rule, with these deceptive signs there is a quickening of the pulse, but not always so. Nor is the diagnosis of the presence of pus excluded by the patient having a normal temperature. One of the largest localised abscesses I have met with was associated with only occasional pyrexia. Pyrexia is in reality a somewhat accidental phenomenon, depending on an excess of thermogenic organisms over others which have been isolated and found to produce a subnormal temperature.

For these suppurative lesions simple drainage was employed in all, and the amount of pus varied from a drachm or two to half a pint. In no case was it possible with any degree of safety to remove the appendix. In only one case has it caused any subsequent trouble. In one case a slight hernia developed in the scar after an interval of about two years. The abscesses in these cases were intra-peritoneal, and were either single or multilocular. Once the position of the abscess was purely lumbar, once lumbar and retrocæcal, and once it occupied the lumbar and pelvic regions. On two occasions the abscesses occupied the lumbar and umbilical regions; and in one patient, for whom a median incision was requisite, the abscess in the umbilical region communicated by a small opening with another large abscess in the pelvis, which was drained by passing a tube through the first abscess into the second. In three cases the pus was not circumscribed, but was escaping freely into the pelvic peritoneum with well-marked signs of generalising peritonitis. The oldest patient was 68 years of age; and the only fatal case was the one previously referred to, in which pneumonia existed before operation, and death resulted from meningitis.

Perhaps the most interesting case, from the point of view of tenacity of life, was that of a young lady who presented symptoms and signs of general peritonitis, and at the first operation pus was seen escaping from behind the cæcum, and dark serous fluid flowed from the general peritoneal cavity. A few days afterwards a large intra-peritoneal abscess was opened *per rectum* — a route employed for special reasons—

and repeatedly repacked with gauze on subsequent days by the same route. Later on, median cœliotomy was requisite to liberate some stinking serous fluid from amongst the intestines. Then pelvic cellulitis developed, an abscess opened into the bladder causing cystitis, and another abscess pointed by way of the left iliac region and discharged through the median wound. Added to these events, the patient had complete intestinal obstruction, with vomiting and absolute constipation for eight days; and when, with considerable pyrexia, she eventually became pulseless, no hope could be reasonably entertained. Yet with large injections of strychnine she began to revive, and she is now a healthy and vigorous young woman. The patient recovered, it is true, but I hope I shall never meet with the like of this case again.

